# Deep Learning and Microbiome Analysis Reveal the Preservation Mechanism of *Cinnamomum cassia* for Strawberry

**DOI:** 10.3390/foods15112005

**Published:** 2026-06-04

**Authors:** Shanxue Jiang, Haishu Sun, Chenyu Zhang, Yihan Zhang

**Affiliations:** School of Light Industry Science and Engineering, Beijing Technology and Business University, Beijing 100048, China; jiangshanxue@btbu.edu.cn (S.J.);

**Keywords:** *Cinnamomum cassia* extract, strawberry preservation, microbiome modulation, deep learning

## Abstract

Strawberry preservation remains a critical challenge due to rapid postharvest microbial spoilage. This study investigated the preservative efficacy of *Cinnamomum cassia* and *Punica granatum* peel aqueous extracts, prepared via spray drying, on strawberries over 5 days of storage, with a specific focus on their regulatory impact on the fruit surface microbiome. Preservation tests demonstrated that the *C. cassia* extract was more effective in reducing visible mold development. High-throughput sequencing revealed that the *C. cassia* treatment reshaped microbial community structures, decreasing the relative abundance of spoilage-associated bacteria and the primary pathogenic fungus *Botrytis* (94.37%), while enriching potentially beneficial or antagonistic genera such as *Sphingobium* (28.72%), *Sphingomonas* (9.52%), and *Cladosporium* (0.62%). Using a probability threshold of 0.7, 121 compounds were identified as potential active candidates from a library of 675 *C. cassia* constituents. These compounds predominantly have a molecular weight between 100 and 250 and are characterized by prevalent functional groups including alkene (49.60%), hydroxyl (38.80%), and benzene rings (36.40%). In vitro antibacterial assays confirmed the inhibitory activity of vanillin and its isomers, validating the reliability of the computational predictions. These findings suggest that the preservative mechanism of *C. cassia* is likely mediated by the collective action of a multi-component matrix that modulates the microecological balance on the fruit surface, rather than the isolated effect of a single compound. This integrated approach provides an effective framework for developing plant-derived preservation strategies by combining microbiome dynamics with machine learning.

## 1. Introduction

Strawberries are highly perishable after harvest due to physiological deterioration and microbial decay, leading to substantial economic losses [1]. This poses a significant challenge for postharvest quality management, creating an urgent need for effective preservation technologies. In recent years, surface treatments have emerged as a promising approach for fruit preservation due to their biodegradability and ability to modulate the fruit microenvironment [2]. For example, a recent study has demonstrated that biodegradable extract-based treatments applied via high-throughput technology can achieve targeted coverage on fruits like avocados, exhibiting remarkable inhibitory effects against harmful microorganisms [3]. This offers a valuable reference for developing novel, safe, and efficient preservation strategies for strawberries.

Plant-derived antimicrobial extracts are important sources for these surface treatments. Among them, extracts from *Cinnamomum cassia* and *Punica granatum* peel are particularly notable for their rich bioactive profiles and broad-spectrum antimicrobial properties. However, elucidating the exact preservation mechanisms of such complex extracts remains challenging [4,5]. Current research primarily focuses on the isolation of single active compounds and their specific antimicrobial mechanisms on microbial cell walls, membranes, DNA, and proteins [6,7]. However, there remains a lack of systematic identification and in-depth analysis of the comprehensive antimicrobial components within complex extracts. These extracts typically contain multiple classes of bioactive compounds (e.g., flavonoids, polysaccharides, phenolic acids, and alkaloids). Relying solely on the evaluation of isolated single compounds fails to capture the collective efficacy of the entire phytocomplex [8,9].

Furthermore, the antimicrobial target of these extracts is rarely a single pathogen. Mature fruits host complex microbial ecosystems, the balance of which plays a critical role in fruit decay [10,11]. While current research tends to focus on in vitro inhibition of specific pathogens or spoilage microorganisms, extract-based treatments actually reshape the entire microbiota on the fruit surface [12]. For instance, it is reported that chitosan-based extract treatment significantly altered the microbial composition on tomato surfaces [13]. Similarly, plant extracts in biopolymer coatings effectively suppress postharvest pathogens and reduce microbial decay in apples [14]. Recent microbial ecology studies suggest that fruit decay is often driven by complex cross-kingdom interactions. Opportunistic spoilage bacteria, such as *Pseudomonas* and *Bacillus* species, can secrete extracellular enzymes that degrade fruit tissues, which facilitates the subsequent invasion of primary fungal pathogens like *Botrytis* [15]. Yet, systematic research integrating the active components of plant extracts, microbial community responses, and fruit decay progression is still lacking.

Bridging the gap between the chemical complexity of plant extracts and their modulatory effects on the fruit microbiome requires advanced analytical tools. Deep learning, a powerful subfield of artificial intelligence, offers a groundbreaking approach to address this challenge [16,17,18]. Unlike conventional methods that rely on the time-consuming isolation and experimental testing of single compounds, deep learning provides a high-throughput computational framework for virtual screening [19]. By learning complex structure-activity relationships, these models can rapidly identify potential antimicrobial candidates from hundreds of constituents within a plant matrix. When combined with microbiome analysis, which identifies the key spoilage microorganisms and pathogens driving fruit decay, deep learning enables a targeted exploration of the extract’s material basis. This integrated strategy provides a novel approach to clarifying how multi-component plant extracts achieve their preservation efficacy through specific microecological regulation.

In this study, the preservative effects of extracts derived from *Cinnamomum cassia* and *Punica granatum* peel on strawberries were investigated, with a focus on their impact on the surface microbiome. To explore the material basis of this activity, deep learning models were developed to predict potential antibacterial compounds, followed by the experimental validation of representative candidates. By integrating microbiome analysis with computational prediction, this study aims to elucidate how plant extracts can preserve fruit by modulating the surface microbial communities. This strategy links extract composition with microbial community dynamics, providing a practical framework for the development of plant-derived fruit preservatives.

## 2. Materials and Methods

### 2.1. Reagents and Materials

*Cinnamomum cassia* and *Punica granatum* peel were obtained from Anhui Junqi Health Technology Co., Ltd., Bozhou, China. Fresh, ripe strawberries were purchased from a local market (Beijing Yonghui Supermarket, Beijing, China) for the preservation study. The bacterial strains *Staphylococcus aureus*, *Escherichia coli*, *Pseudomonas aeruginosa*, and *Bacillus subtilis* were provided by the Beijing Biological Conservation Center (Beijing, China). Vanillin, isovanillin, and *o*-vanillin were supplied by Shanghai Macklin Biochemical Co., Ltd., Shanghai, China. All chemicals and reagents used in this study were of analytical grade.

### 2.2. Preparation of Cinnamomum cassia and Punica granatum Peel Extracts

*Cinnamomum cassia* and *Punica granatum* peel were first pulverized into a fine powder. To ensure food safety, eliminate the risk of organic solvent residues on fruit surfaces, and align with environmentally friendly food processing standards, pure water was selected as the sole extraction solvent [20,21,22]. Each powdered sample was then mixed with pure water at a ratio of 1:8 (*w*/*v*) and shaken at 110 rpm and 50 °C for 3 h. Following extraction, a 5% (*w*/*v*) polyacrylamide (PAM) solution was added to the crude extract. PAM is a flocculant widely utilized in food processing such as sugar juice clarification. It was employed here specifically to aggregate and remove insoluble plant cell debris and colloidal impurities [23]. The mixture was stirred for 40 min and subsequently centrifuged at 4000 rpm for 5 min, after which the supernatant was collected. The supernatant was then spray-dried to obtain the dry extract powder. Finally, each dry extract was dissolved in pure water to prepare a 3% (*w*/*v*) antimicrobial solution for subsequent experiments.

### 2.3. Antimicrobial Tests

The antimicrobial activity of the *Cinnamomum cassia* and *Punica granatum* peel extracts was investigated via the agar well diffusion method. Briefly, 1 mL of bacterial suspension was uniformly spread on agar plates. A well (6 mm in diameter) was made in the center of the agar plate, and the plug was carefully removed. Subsequently, 3% (*w*/*v*) extract solution was introduced into the well, and the plates were incubated at 37 °C for 12 h. After incubation, the inhibition zones around each well were measured to evaluate antibacterial activity. All experiments were performed in triplicate.

### 2.4. Strawberry Preservation Tests

An immersion method was employed to apply the preservative plant extracts onto the strawberry surfaces. Briefly, each group of strawberries (n = 6) was immersed in the respective 3% (*w*/*v*) extract treatment solutions (*Cinnamomum cassia* extract or *Punica granatum* peel extract) for 1 min. Following immersion, the strawberries were air-dried under a fume hood at room temperature until no visible moisture droplets remained on the fruit surfaces. Strawberries treated with ultrapure water served as the control group. All treated strawberries were then stored in identical plastic containers at room temperature. The visual appearance, with a focus on mold development and spoilage, was observed and recorded daily throughout the storage period.

### 2.5. DNA Extraction and High-Throughput Sequencing

To evaluate the effect of the extract treatments on the strawberry surface microbiome, microbial samples were collected on day 5 of storage. Three biological replicates were established for each treatment group (Control, *Cinnamomum cassia* extract, and *Punica granatum* peel extract), with each replicate comprising the surface microbiome from an individual fruit. The specific procedures for surface microorganism collection, DNA extraction, and PCR amplification were executed in accordance with previously established protocols [22]. Briefly, total microbial genomic DNA was extracted from the fruit surface eluate using the E.Z.N.A.^®^ soil DNA Kit (Omega Bio-Tek, Norcross, GA, USA). The V3–V4 region of the bacterial 16S rRNA gene and the fungal ITS region were amplified and subsequently sequenced on an Illumina MiSeq PE250 platform. Raw sequence data processing, quality control, as well as bioinformatic and statistical analyses were performed on the Majorbio Cloud Platform (https://www.majorbio.com).

### 2.6. Model Training and Identification of Antibacterial Components

**Figure 1:**
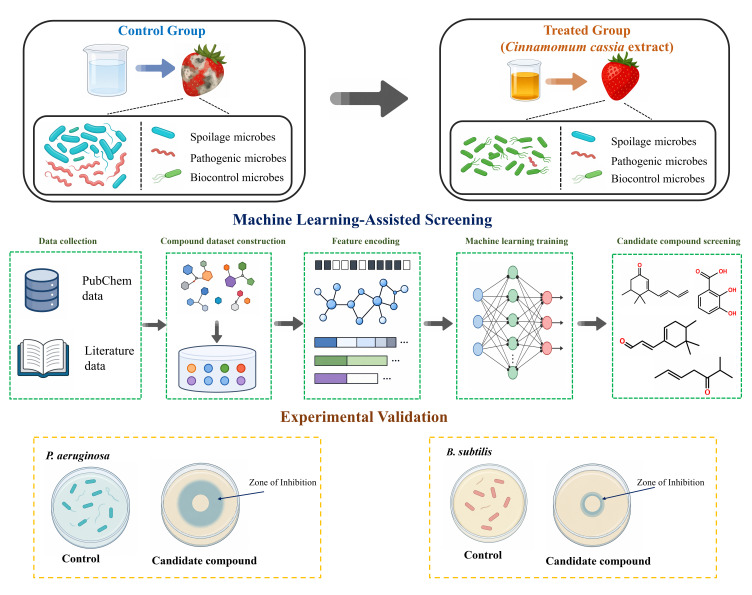
Schematic illustration of the integrated methodology, combining microbiome analysis and machine learning, to investigate the preservation mechanism of *Cinnamomum cassia* extract on strawberries.

To identify the specific compounds responsible for the preservative efficacy of *Cinnamomum cassia* extract, two predictive models for antibacterial activity against *P. aeruginosa* and *B. subtilis* were constructed and evaluated (Figure 1). Standardized datasets of known active (labeled as 1) and inactive (labeled as 0) compounds in SMILES format were collected for *P. aeruginosa* and *B. subtilis* to train the two distinct models (see Supplementary Data S1 and S2). Prior to model training, the Simplified Molecular Input Line Entry System (SMILES) strings of all compounds were preprocessed and canonicalized using the RDKit toolkit in Python 3.8 to ensure structural consistency.

Model training was performed using Molactivity, a molecular activity prediction toolkit developed by our group and can be installed as a python package using the commond “pip install molactivity” [24]. For each of the two target bacteria, the collected dataset was split into a training set and an evaluation set, with six parallel models trained per bacterium. To ensure an unbiased assessment of model accuracy, no SMILES were shared between the two sets. Due to the limited amount of data, after the evaluation was completed, the training set and the evaluation set were merged into a new, combined training set, which was then used to train the final models. To ensure the reliability of the results, three independent models were trained finally under identical conditions for each target bacterium. The datasets for deep learning model training and evaluation are summarized in Table 1.

Model performance was comprehensively evaluated using five statistical metrics: accuracy, precision, recall, F1-score, and the area under the ROC curve (AUC). Ultimately, the optimal models were applied to screen a library of 675 chemical constituents reported in *Cinnamomum cassia*, whose SMILES structures were retrieved from PubChem and ChemSpider (see Supplementary Data S1), to identify candidates with high probabilities of possessing antibacterial activity. To experimentally verify the model predictions, representative compounds were selected for antimicrobial tests using the same agar well diffusion method described in Section 2.3.

### 2.7. Statistical Analysis

All experimental assays, including the antimicrobial tests and preservation evaluations, were performed in triplicate. The numerical data were expressed as the mean ± standard deviation (SD). The high-throughput sequencing data of the microbial communities were processed and statistically analyzed on the Majorbio Cloud Platform.

## 3. Results and Discussion

### 3.1. Preservative Efficacy of Cinnamomum cassia and Punica granatum Peel Extracts on Strawberries

**Figure 2:**
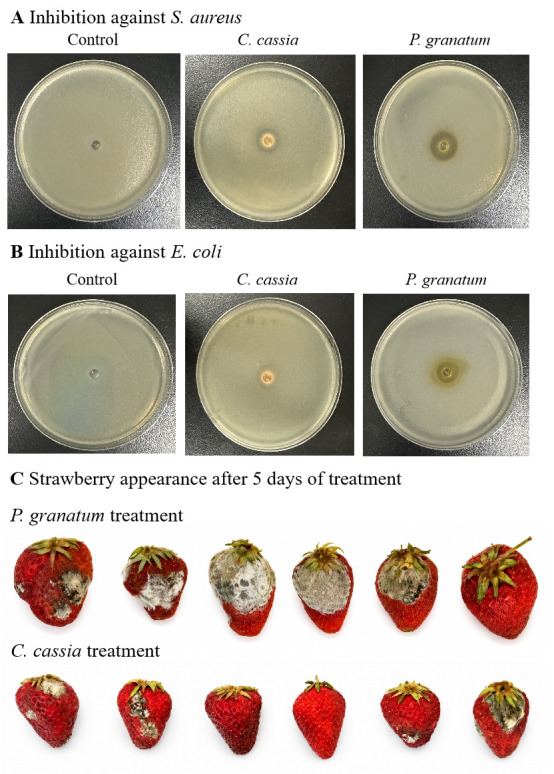
Antibacterial activities and preservative efficacy of *Cinnamomum cassia* and *Punica granatum* peel aqueous extracts. (**A**) In vitro antibacterial activities against *Staphylococcus aureus*. (**B**) In vitro antibacterial activities against *Escherichia coli*. (**C**) Visual appearance of strawberries after 5 days of treatment.

The postharvest spoilage of strawberries is a complex biological process driven by a diverse array of microorganisms. To initially assess the broad-spectrum antimicrobial potential of *Cinnamomum cassia* and *Punica granatum* peel extracts, *Staphylococcus aureus* and *Escherichia coli* were utilized as standard Gram-positive and Gram-negative indicator strains. As shown in Figure 2A,B, both aqueous extracts exhibited clear antibacterial activity in vitro. Based on these screening results, the extracts were subsequently applied to fresh strawberries to evaluate their practical preservative efficacy.

Macroscopic observations after 5 days of storage revealed distinct differences in preservative efficacy among the treatments (Figure 2C). Strawberries treated with the *C. cassia* aqueous extract showed minimal visible mold development. In contrast, the control group exhibited the most severe decay [22]. The *P. granatum* peel treatment resulted in an intermediate level of mold growth, outperforming the control but remaining visibly less effective than the *C. cassia* treatment.

Interestingly, although the *P. granatum* extract demonstrated strong in vitro inhibition against these specific indicator bacteria, its moderate performance on the actual fruit suggests that in vitro assays against isolated bacterial strains cannot fully predict real-world preservation outcomes. Fruit spoilage in practice is dictated by complex microbial ecosystems rather than single pathogens. While typical postharvest studies often include a broader range of physicochemical metrics (e.g., firmness and weight loss), this study utilized macroscopic visual decay as the primary phenotypic marker to bridge the gap with the underlying microbiological shifts. Therefore, to understand the mechanism behind the superior protection provided by *C. cassia*, it is necessary to examine how these treatments modulated the native microbial communities on the strawberry surface, as detailed in Section 3.2 and Section 3.3.

### 3.2. Effects of Cinnamomum cassia and Punica granatum Peel Treatment on the Bacterial Community on Strawberry Surfaces

**Figure 3:**
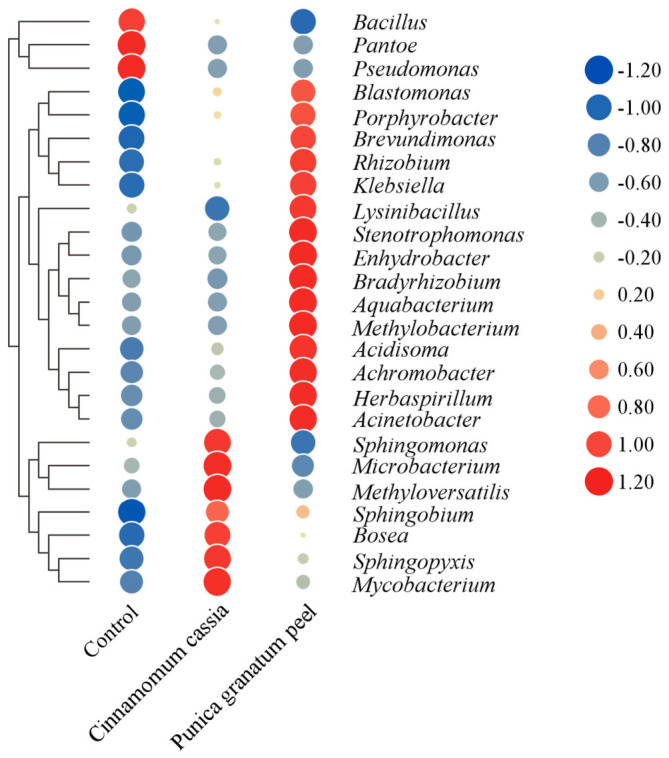
Relative abundance of bacterial communities at the genus level on strawberry surfaces following treatment with *Cinnamomum cassia* and *Punica granatum* peel extracts. The color gradient and bubble size represent the standardized relative abundance (Z-score) of each bacterial genus across different groups.

To understand the microbiological changes associated with the observed visual decay, the bacterial communities on the strawberry surfaces were analyzed via 16S rRNA gene sequencing. As shown in Figure 3, both *C. cassia* and *P. granatum* peel treatments altered the bacterial composition at the genus level compared to the control.

Specifically, both treatments showed inhibitory effects against certain spoilage-associated genera. The relative abundance of *Bacillus*, a genus containing species known to secrete extracellular hydrolases that contribute to food decay [25], decreased in both treatment groups. Similarly, *Pseudomonas*, a common spoilage bacterium [26], was reduced to 0.02% in both treatments. Furthermore, the relative abundance of *Pantoea*, which includes species with potential pathogenicity toward plants [27], decreased to 0.02% in the *C. cassia* group and was rarely detected in the *P. granatum* group.

However, the treatments showed different effects on other taxa, which might relate to their varying preservation efficacy. For instance, *Lysinibacillus* can also produce extracellular hydrolases and proteases linked to food decay [28]. Interestingly, while its relative abundance was 8.33% in the *C. cassia* treatment group, it increased to 41.03% in the *P. granatum* peel treatment group. The higher abundance of this potential spoilage-associated genus might be one reason why the *P. granatum* peel extract was less effective in preserving the strawberries compared to *C. cassia*.

Conversely, the relative abundance of some potentially beneficial bacterial genera increased following the extract treatments. *Sphingobium*, generally considered a beneficial microorganism [29], increased to 28.72% and 23.73% in the *C. cassia* and *P. granatum* peel treatment groups, respectively. Another genus, *Sphingomonas*, which has been reported to possess plant growth-promoting properties and antagonistic activity against fungal pathogens [30], showed a different trend. It increased to 9.52% in the *C. cassia* group, but remained at 1.17% in the *P. granatum* peel group. The combined observation of spoilage bacteria suppression and the potential enrichment of beneficial taxa like *Sphingomonas* provides a reasonable microbiological context for the superior preservative efficacy of the *C. cassia* treatment.

### 3.3. Effects of Cinnamomum cassia and Punica granatum Peel Treatment on the Fungal Community on Strawberry Surfaces

**Figure 4:**
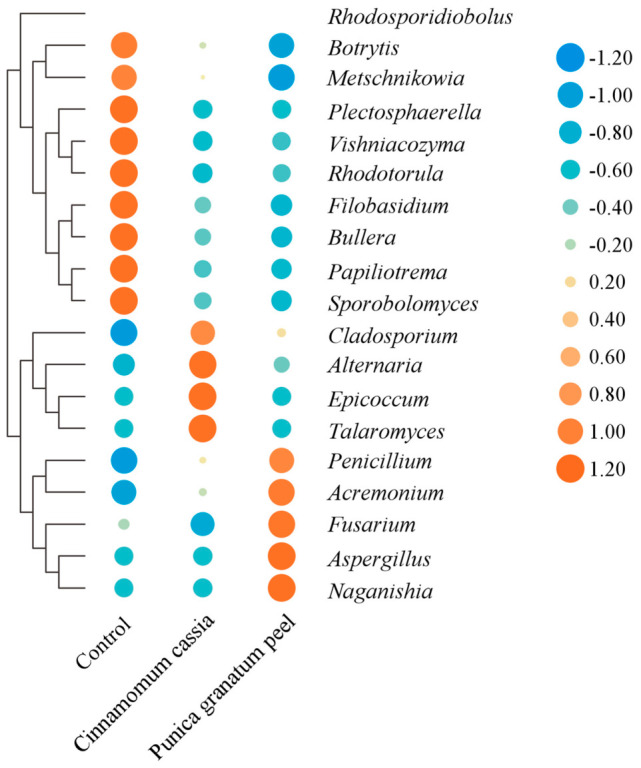
Relative abundance of fungal communities at the genus level on strawberry surfaces following treatment with *Cinnamomum cassia* and *Punica granatum* peel extracts. The color gradient and bubble size represent the standardized relative abundance (Z-score) of each fungal genus across different groups.

To evaluate the fungal community dynamics, ITS region sequencing was performed. As illustrated in Figure 4, *Botrytis* was the most abundant genus in the control group. This observation aligns with existing literature, as *Botrytis* is widely recognized as a common pathogen associated with strawberry gray mold and postharvest decay [31,32].

Following the application of the plant extracts, the relative abundance of *Botrytis* was 94.37% in the *C. cassia* treatment group and 90.99% in the *P. granatum* peel treatment group. While *Botrytis* remained the major fungal taxon across all samples, these slight reductions compared to the control suggest a potential, albeit limited, inhibitory effect on its growth. Concurrently, the relative abundance of *Cladosporium* displayed an upward trend, increasing to 0.62% and 0.44% in the *C. cassia* and *P. granatum* peel groups, respectively. As certain *Cladosporium* species have been reported to exhibit antagonistic activity against phytopathogens [33], this subtle increase might contribute to a competitive microenvironment on the fruit surface.

When analyzing these fungal shifts alongside the macroscopic preservation outcomes (Section 3.1), an interesting dynamic was observed. The *P. granatum* peel extract showed a slightly lower abundance of *Botrytis* compared to *C. cassia*, yet its overall preservation efficacy was less effective. This observation suggests that attributing fruit spoilage solely to changes in a single pathogenic fungal genus may be insufficient. Integrating these fungal results with the bacterial community data (Section 3.2) provides a broader perspective. While the *P. granatum* peel treatment was associated with a slightly lower *Botrytis* abundance, it also coincided with a notable increase in the potential spoilage bacterium *Lysinibacillus* (reaching 41.03%). In contrast, the *C. cassia* treatment showed a different microbial profile, moderately suppressing the main fungal pathogen and maintaining a higher abundance of potential fungal antagonists (*Cladosporium*), while simultaneously exhibiting lower levels of certain spoilage bacteria (*Bacillus* and *Pseudomonas*) and higher levels of potentially beneficial taxa (*Sphingomonas*).

These combined microbiome analyses suggest that the preservation effect of the *C. cassia* extract may be related to a broader modulation of both bacterial and fungal communities. Maintaining this relative microecological balance could be a contributing factor to the reduced visual decay observed in the *C. cassia* treatment.

### 3.4. Model Training and Screening of Antibacterial Components

Given the superior preservative efficacy of *C. cassia* extract in the strawberry preservation test, subsequent deep learning screening and active component validation were directed toward *C. cassia* to elucidate its underlying preservative mechanism.

Based on the microbiome analysis in Section 3.2 and Section 3.3, the preservation mechanism of *C. cassia* appears to involve maintaining microecological balance, partially by suppressing key opportunistic spoilage bacteria alongside fungal pathogens. To further investigate the material basis of this antimicrobial effect, two predictive models were constructed targeting *Pseudomonas aeruginosa* and *Bacillus subtilis*. These species were selected as representative strains for the *Pseudomonas* and *Bacillus* genera, which were observed to be inhibited by the *C. cassia* treatment on the strawberry surfaces. While developing a direct predictive model against the dominant fungal pathogen (*Botrytis*) would be ideal, the current scarcity of large-scale, standardized molecular activity datasets for phytopathogenic fungi makes this computationally challenging. Previous studies have indicated that these two species are prevalent and significant contributors to food spoilage, thereby justifying their representativeness in this study [34,35].

The high prevalence of these functional groups, particularly phenolic hydroxyls and benzene rings, aligns well with the established antimicrobial mechanisms of natural plant extracts. The lipophilic nature of the benzene ring facilitates the partition of these molecules into the bacterial lipid bilayer, thereby increasing membrane permeability [36]. Concurrently, the hydroxyl groups can cause direct damage to the bacterial cell membrane, leading to the leakage of essential intracellular constituents and eventual cell death [37]. By identifying a cluster of diverse compounds sharing these specific sub-structures, the deep learning model implicitly captures the chemical rules governing plant-derived antibacterials. Furthermore, this structural diversity within the extract exerts a multi-target inhibitory effect, making it significantly more difficult for spoilage bacteria to develop resistance compared to conventional single-target synthetic preservatives [38].

**Figure 5:**
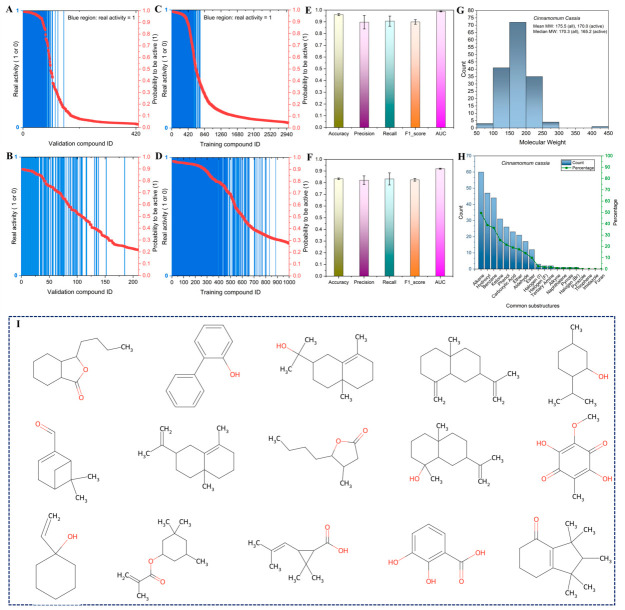
Predictive performance of the deep learning models and chemical profiling of the identified active compounds. Distribution of true activity labels and predicted probabilities for the evaluation sets of the anti-*Pseudomonas aeruginosa* (**A**) and anti-*Bacillus subtilis* (**B**) models. Distribution of true activity labels and predicted probabilities for the final training sets of the anti-*Pseudomonas* aeruginosa (**C**) and anti-*Bacillus subtilis* (**D**) models. Performance evaluation metrics for the anti-*Pseudomonas aeruginosa* (**E**) and anti-*Bacillus subtilis* (**F**) models. Chemical features of the predicted antimicrobial compounds from *Cinnamomum cassia*, including molecular weight distribution (**G**), top functional substructures (**H**), and chemical structures of representative candidates (**I**).

As shown in Figure 5A, the anti-*Pseudomonas aeruginosa* models achieved robust performance when evaluated on an independent dataset. In contrast, the anti-*Bacillus subtilis* models (Figure 5B) did not perform as well as the anti-*Pseudomonas aeruginosa* models, probably because the dataset for *P. aeruginosa* was substantially larger than that for *B. subtilis.* To maximize the utility of the available data, after the evaluation was completed, the evaluation set was merged with the training set to retrain the final models. The performance of these retrained models on the combined training data is presented in Figure 5C–F. The results indicated that both models can effectively distinguish between active and inactive compounds, although the anti-*P. aeruginosa* models consistently outperformed the anti-*B. subtilis* models. Subsequently, these models were applied to screen a library of 675 chemical constituents reported in *C. cassia*. Using a probability threshold of 0.7, a total of 121 compounds were predicted to possess potential antibacterial activity against both *B. subtilis* and *P. aeruginosa* simultaneously (Supplementary Data S2).

These 121 predicted compounds exhibited a typical low molecular weight distribution, predominantly ranging between 100 and 250 (Figure 5G). The identification of over 120 potential antibacterial candidates suggests that the antimicrobial properties of the *C. cassia* extract are likely not dependent on a single highly active constituent, but rather arise from the synergistic action of a multi-component matrix. Further structural analysis of these predicted active compounds revealed a prevalence of specific functional groups, including alkene (49.60%), hydroxyl (38.80%), and benzene rings (36.40%), followed by ketone, phenol, and carboxylic acid groups (Figure 5H). The recurring cyclic scaffolds among the top predicted compounds provide a structural reference for understanding the broad bioactivity of the extract and guide the selection of representative candidates for subsequent in vitro validation (Figure 5I).

### 3.5. Experimental Validation of Vanillin and Its Isomers

**Figure 6:**
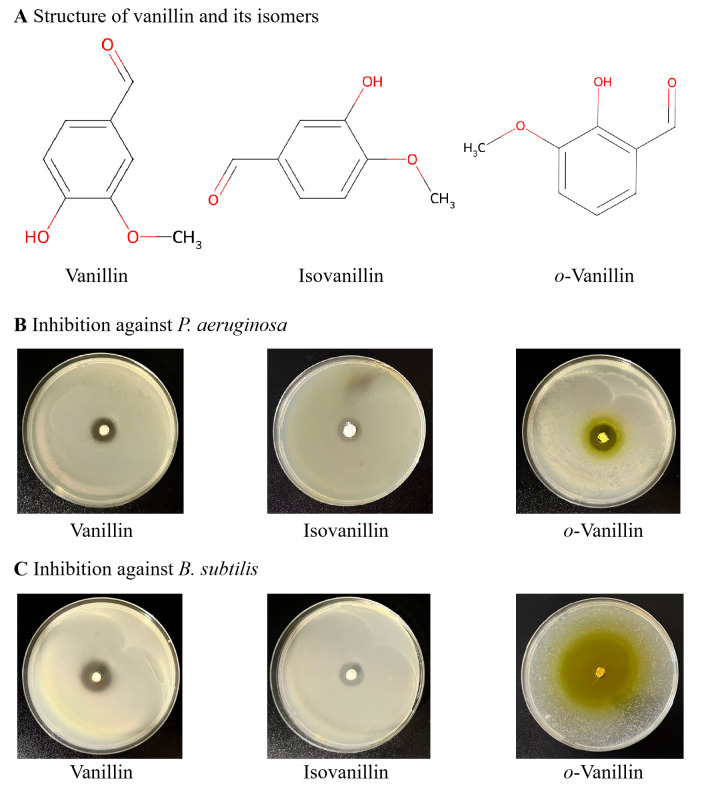
(**A**) Chemical structures of vanillin and its isomers (isovanillin and *o*-vanillin). In vitro antibacterial activities of these compounds against (**B**) *Pseudomonas aeruginosa* and (**C**) *Bacillus subtilis*, evaluated using the agar well diffusion method.

To verify the deep learning model’s ability to identify antibacterial compounds, vanillin and two of its isomers (isovanillin and *o*-vanillin) were selected for in vitro assays based on their computational screening rankings and commercial availability. These compounds share the same molecular formula (C_8_H_8_O_3_) and core functional groups (benzene ring, phenolic hydroxyl, aldehyde, and methoxy groups) (Figure 6A), aligning well with the predicted active structural features. Importantly, these assays were designed to validate the reliability of the computational predictions at the molecular level, rather than to directly predict in vivo preservation efficacy on fruit, which involves more complex multi-component interactions.

As shown in Figure 6B,C, all three compounds exhibited distinct zones of inhibition against *B. subtilis* and *P. aeruginosa* in the agar well diffusion assay. These results provide preliminary validation for the reliability of the computational screening methodology in identifying active constituents from complex plant extracts. Furthermore, the comparable antibacterial activities among the three isomers indicate that positional variations in the functional groups on the benzene ring do not significantly reduce their inhibitory efficacy, providing a fundamental reference for understanding the structure-activity relationship of such active components.

While these compounds demonstrated strong in vitro antibacterial activity, such activity does not always translate directly to practical preservation effectiveness. As evidenced by our *P. granatum* peel extract findings (Section 3.1), strong in vitro inhibition may not prevent secondary spoilage if the treatment disrupts the natural competitive balance and facilitates the overgrowth of specific spoilage bacteria (e.g., *Lysinibacillus*) [10,39]. Therefore, the actual preservation of strawberries by *C. cassia* is unlikely to rely solely on these few validated molecules. Instead, numerous minor constituents in the plant extract likely exert significant synergistic interactions [40]. Non-antimicrobial components can improve the aqueous solubility of highly active lipophilic molecules, facilitate their uniform distribution across the hydrophilic fruit epidermis, or assist in penetrating the protective extracellular matrices formed by surface microbes [41]. Consequently, the isolated application of vanillin isomers in planta may not fully replicate the comprehensive efficacy of the crude extract, highlighting that the overall preservative effect of *C. cassia* is driven by the collective modulation of the microbial community by the entire phytocomplex [42].

Finally, several limitations regarding this validation step should be noted to guide future research. First, the agar well diffusion method serves primarily as a qualitative validation of the model’s binary predictions (active vs. inactive). Future studies must incorporate rigorous quantitative evaluations, including minimum inhibitory concentration (MIC) and minimum bactericidal concentration (MBC) determinations, to fully elucidate the specific inhibitory potencies of these constituents. Second, bridging the gap between molecular-level predictions and practical outcomes requires systematic in planta experiments to assess compound stability, tissue penetration, and interactions with the native microbiome under realistic storage conditions. Lastly, while these active compounds were identified through computational screening based on existing databases for *C. cassia*, the experimental confirmation of their exact presence and concentrations in the current aqueous extract was not performed. Future research will focus on the targeted metabolomic characterization (e.g., via LC-MS/GC-MS) of the extract to fully substantiate these computational predictions.

## 4. Conclusions

This study investigated the preservative effects of *Cinnamomum cassia* and *Punica granatum* peel extracts on strawberries by combining microbiome analysis with deep learning. Preservation tests showed that *C. cassia* extract outperformed *P. granatum* peel extract. Microbiome analysis revealed that the superior efficacy of *C. cassia* was associated with its regulation of the surface microbial community, characterized by the suppression of spoilage-associated bacteria (e.g., *Bacillus* and *Pseudomonas*) and the primary fungal pathogen (*Botrytis*), alongside the enrichment of potentially beneficial genera (e.g., *Sphingomonas* and *Cladosporium*). Furthermore, deep learning-based screening identified 121 potential antibacterial compounds from *C. cassia*, featuring functional groups such as alkene, hydroxyl, and benzene rings. The in vitro validation of vanillin and its isomers confirmed the reliability of the computational predictions. Overall, the integrated approach presented here provides an effective framework for understanding and developing plant-derived preservation strategies.

## Figures and Tables

**Table 1:** Description of datasets for deep learning model training and evaluation.

Dataset	Anti-*Pseudomonas aeruginosa* Models	Anti-*Bacillus subtilis* Models
Labelled as 1 (initial training)	468	383
Labelled as 0 (initial training)	2092	404
Labelled as 1 (initial evaluation)	100	86
Labelled as 0 (initial evaluation)	328	124
Labelled as 1 (all, final training)	568	469
Labelled as 0 (all, final training)	2420	528

## Data Availability

The original contributions presented in this study are included in the article/Supplementary Materials. Further inquiries can be directed to the corresponding author.

## Supplementary Materials

The following supporting information can be downloaded at: https://www.mdpi.com/article/10.3390/foods15112005/s1, Data S1: anti-*P. aeruginosa* models; Data S2: anti-*B. subtilis* models.
